# Using Normalization Process Theory in feasibility studies and process evaluations of complex healthcare interventions: a systematic review

**DOI:** 10.1186/s13012-018-0758-1

**Published:** 2018-06-07

**Authors:** Carl R. May, Amanda Cummings, Melissa Girling, Mike Bracher, Frances S. Mair, Christine M. May, Elizabeth Murray, Michelle Myall, Tim Rapley, Tracy Finch

**Affiliations:** Faculty of Public Health and Policy, London School of Hygiene and Tropical Medicine, London, UK

**Keywords:** Normalization Process Theory, Complex interventions, Implementation research, Process evaluation, Systematic review

## Abstract

**Background:**

Normalization Process Theory (NPT) identifies, characterises and explains key mechanisms that promote and inhibit the implementation, embedding and integration of new health techniques, technologies and other complex interventions. A large body of literature that employs NPT to inform feasibility studies and process evaluations of complex healthcare interventions has now emerged. The aims of this review were to review this literature; to identify and characterise the uses and limits of NPT in research on the implementation and integration of healthcare interventions; and to explore NPT’s contribution to understanding the dynamics of these processes.

**Methods:**

A qualitative systematic review was conducted. We searched Web of Science, Scopus and Google Scholar for articles with empirical data in peer-reviewed journals that cited either key papers presenting and developing NPT, or the NPT Online Toolkit (www.normalizationprocess.org). We included in the review only articles that used NPT as the primary approach to collection, analysis or reporting of data in studies of the implementation of healthcare techniques, technologies or other interventions. A structured data extraction instrument was used, and data were analysed qualitatively.

**Results:**

Searches revealed 3322 citations. We show that after eliminating 2337 duplicates and broken or junk URLs, 985 were screened as titles and abstracts. Of these, 101 were excluded because they did not fit the inclusion criteria for the review. This left 884 articles for full-text screening. Of these, 754 did not fit the inclusion criteria for the review. This left 130 papers presenting results from 108 identifiable studies to be included in the review. NPT appears to provide researchers and practitioners with a conceptual vocabulary for rigorous studies of implementation processes. It identifies, characterises and explains empirically identifiable mechanisms that motivate and shape implementation processes. Taken together, these mean that analyses using NPT can effectively assist in the explanation of the success or failure of specific implementation projects. Ten percent of papers included critiques of some aspect of NPT, with those that did mainly focusing on its terminology. However, two studies critiqued NPT emphasis on agency, and one study critiqued NPT for its normative focus.

**Conclusions:**

This review demonstrates that researchers found NPT useful and applied it across a wide range of interventions. It has been effectively used to aid intervention development and implementation planning as well as evaluating and understanding implementation processes themselves. In particular, NPT appears to have offered a valuable set of conceptual tools to aid understanding of implementation as a dynamic process.

**Electronic supplementary material:**

The online version of this article (10.1186/s13012-018-0758-1) contains supplementary material, which is available to authorized users.

## Background

Implementation theories are useful. They provide explanations for relevant phenomena, propose important research questions and frame the collection and analysis of data [1]. These explanations are generalizable and facilitate comparative studies. Implementation researchers now have a wide range of useful theoretical tools at their disposal [2–4]. Normalization Process Theory (NPT) [5–10] is one of these. It identifies, characterises and explains mechanisms that have been empirically demonstrated to motivate and shape implementation processes and affect their outcomes. This paper presents a systematic review of studies of healthcare interventions informed by NPT.

### What is NPT and what does it do?

NPT is a theory of implementation that focuses on what people—both individuals and groups—do rather than what they believe or intend, and it has been built up from studies of practice in many different healthcare systems. This means that it focuses attention on aspects of individual and collective behaviour shown to be important in empirical studies of implementation processes. The development of NPT first involved the iterative development of a robust generic theory of implementation [5–9, 11, 12]. From this, tools were developed to assist implementation practitioners and researchers [13–16] in thinking through and measuring important elements of implementation processes. In its most recent iteration, we have shown how the basic mechanisms characterised in NPT function as self-organising mechanisms in complex adaptive social systems [10]. Theory development in NPT has been iterative, with three phases of development around practical questions.

1. Objects: How are components of complex interventions operationalised by their users? In the first iteration of the theory—the Normalization Process Model (NPM) [5, 6]—we identified the importance of collective action in routinely incorporating complex interventions into everyday practice. We showed how collective action was organised around interactions between users and the properties of intervention components.
2. Agents: What is the work of implementing a new technique, technology or organisational intervention? In the second iteration of the theory—Normalization Process Theory (NPT) [7, 8]—we characterised mechanisms (coherence, cognitive participation, collective action and reflexive monitoring) that motivate and shape implementation processes and explained their operation.
3. Contexts: How are structural and cognitive resources for implementation mobilised and what mechanisms lead to variations in implementation processes over time and between settings? In the most recent iteration of the theory—Extended Normalization Process Theory (ENPT) [9, 10]—we pointed to the dynamic role of implementation contexts in the mobilisation and negotiation of implementation processes.

Underpinning these practical questions is one that is fundamental to the social and behavioural sciences—and especially to behavioural economics, sociology and social psychology—which is *how can we best understand the dynamics of human agency under conditions of constraint* [10]? The important implication of this question is that well-designed, theoretically informed studies in implementation research actually offer opportunities for basic investigations in the social sciences.

### The purpose of this review

A review by McEvoy et al. [17], published in 2014, provided a qualitative synthesis of 29 early and heterogeneous studies in which NPT was used. It drew attention to a positive response from healthcare researchers to the theory, but it also made three important critical points about the emerging NPT literature. McEvoy et al. [17] pointed to the ways that early studies using NPT did little work to justify the choice of theory, called for the prospective application of NPT to data analysis and collection and stressed the importance of moving beyond single stakeholder perspectives.

In the period since McEvoy et al.’s review [17], studies using NPT have proliferated. There are now a large number of protocols, empirical studies and reviews in which NPT plays a role. Importantly, a large number of NPT studies have now been completed by groups who are independent of the theory’s architects. It is therefore an opportune time to undertake a qualitative systematic review that will (i) identify and characterise the uses and limits of NPT in research on the implementation and integration of healthcare interventions and (ii) explore NPT’s contribution to understanding the dynamics of these processes.

## Methods

### Systematic citation searches

As the aim of this qualitative systematic review was to identify the uses of NPT in research on the implementation and integration of health care interventions since the publication of the first iteration of the theory in 2006, our search strategy was focused on citations. Following Kirk et al.’s review of reports of the Consolidated Framework for Implementation Research [18], we searched two bibliographic two databases (Scopus and Web of Science), and a search engine (Google Scholar), to search for citations of key papers that developed or expounded the main constructs of NPT [5–9, 11, 12], papers that developed NPT related methods or tools [13–15] and citations of the NPT web-enabled on-line toolkit (www.normalizationprocess.org) [16]. Searches were conducted by AC, MG, CRM, MM and TLF. The sensitivity of the search strategy was tested against a database of studies using NPT that had been collected by three of the co-authors (CRM, TR, TF). All studies already known to use NPT at December 2015 were identified by the first round of systematic searches. Searches were initially undertaken in June 2015 and were updated in December 2015, August 2016 and March 2017. A final search was undertaken in December 2017.

### Inclusion and exclusion criteria

We included the following: peer-reviewed English language journal articles reporting empirical research on the implementation of healthcare interventions, in which NPT was the primary analytic framework (applied either prospectively in study design and data collection, or retrospectively in the interpretation of already collected data) and which were undertaken in any healthcare setting. We define an empirical paper as one that contains evidence of data collection and analysis. We included studies that used any method of empirical investigation (qualitative, quantitative, and mixed methods).

We excluded the following: papers in which NPT was used as a framework for systematic reviews or meta-syntheses; papers solely on patient and caregiver experiences; papers in which NPT was not the primary analytic theory; editorials, theory and methods discussion papers; papers containing passing references to NPT; study protocols; papers describing work undertaken in settings other than healthcare; and papers published in languages other than English. We also excluded theses or dissertations, books and book chapters, conference proceedings and abstracts. We did not exclude papers on the grounds of methodological quality. We already knew that the literature ranged from student projects through to process evaluations in large and well-designed clinical trials in which NPT informed all activities from design through process evaluation and follow-up, to interpretation of trial outcomes. All studies were equally interesting to us, because we were searching for information about the way in which the theory was used rather than the summative results of NPT analyses.

### Screening

Screening started with an assessment of citations and abstracts’ relevance by reviewers who had not been involved in the development of NPT (AC and MM). Reports that met eligibility criteria were obtained in full text. Full-text papers were screened by pairs of reviewers (AC with MM or CRM; MB with CRM; or CRM and TF) working independently of each other. Full-text screening consisted of identifying papers where NPT was clearly the analytic framework for an empirical study. Because no ‘one best way’ to operationalise NPT and its constructs has been prescribed, we did not apply judgments about this to screened papers. This meant that screening involved a simple Yes/No question, and references were sorted within Endnote Libraries accordingly.

### Data extraction

We developed an extraction instrument, (see Additional file 1: Appendix 1). Data were extracted by all authors except CMM, FSM and EM. To avoid conflicts of interest, authors or co-authors of included papers were not involved in extracting data from those papers. Data were extracted on authors, year of publication, health care problem addressed, study type and methods, data collection procedures, how NPT was used in the study and whether this had been pre-specified in the study protocol. We looked for data on whether and how NPT had contributed to understanding the dynamics of the processes of implementation and integration, and for authors’ views about the limitations of NPT in terms of both its scope (what the theory explains) and application (what happens when researchers use the theory). As this was a qualitative review, we included data from both the results and discussion sections of included papers.

### Data analysis

Coding and initial interpretation work was undertaken using the extraction instrument. To ensure consistency, CRM and TLF jointly checked coding on 75/130 of included papers, and CRM and CMM jointly checked categorisation of all included papers. The analysis aimed (i) to identify and characterise the uses and limitations of NPT in research on the implementation and integration of healthcare interventions and (ii) to explore NPT’s contribution to understanding the dynamics of these processes. Hence, we started by describing how NPT had been used and subsequently analysed the data to explore the ways that mechanisms defined by NPT have been revealed to operate. We sought to understand the relative importance of specific NPT constructs across different settings (core processes and mechanisms) and differences that seemed to apply in relation to different intervention types and healthcare systems (contingent processes and mechanisms).

### Public registration of the review

PROSPERO deemed this review ineligible for public registration on the grounds that NPT was not a healthcare intervention.

## Results

### Search results

**Fig. 1:**
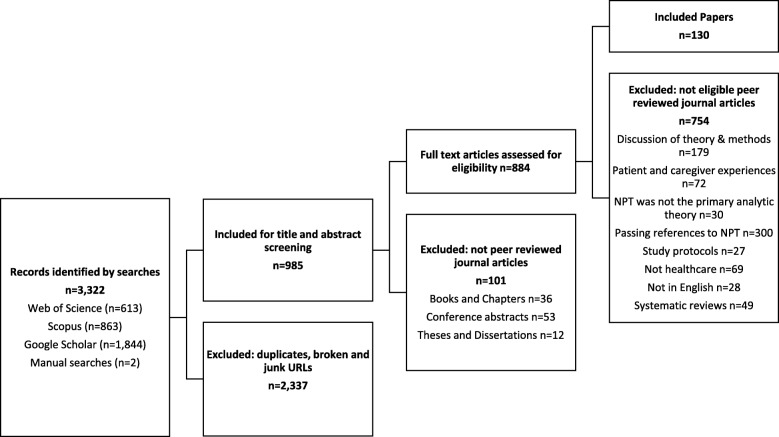
PRISMA flowchart

Searches revealed 3322 citations. In Fig. 1, we show that after eliminating 2337 duplicates and broken or junk URLs, 985 were screened as titles and abstracts. Of these, 101 were excluded because they did not fit the inclusion criteria for the review. This left 884 articles for full-text screening. Of these, 754 did not fit the inclusion criteria for the review. This left 130 papers presenting results from 108 identifiable studies to be included in the review.

### Types of studies

In this review, 130 papers reported the application of NPT in 108 identifiable studies. Included articles presented both controlled (*n* = 26) and uncontrolled (*n* = 82) studies.

In Table 1, we show that NPT was employed in 26 controlled studies—mainly complex intervention trials—and these generated 40/130 (30.8%) articles [19–58]. These included an intervention design study (*n* = 1), feasibility studies (*n* = 5), process evaluations (*n* = 19) and retrospective documentary analyses (*n* = 1), embedded in complex intervention trials. Three of these studies used mixed methods, and one [55] was a survey. The remainder (*n* = 22) all used qualitative methods.

**Table 1:** Controlled studies using NPT as their analytic framework

First author/first paper	Country of origin	Theory frame	Research problem	Evidence base cited to support intervention	Use of NPT specified in protocol	NPT study type	Data collected	Application of NPT to data	Factors leading to intervention success or failure	Differences between categories of participants	Differences between settings
1. Ballinger et al. [19]	UK	NPT	Stroke rehabilitation	Systematic review [149]	–	Process evaluation	Qualitative	Retrospective	Yes	No	No
2. Bamford et al. [20]	UK	NPT	New professional roles in dementia care	Systematic review [150]	Yes	Process evaluation	Qualitative	Prospective	Yes	Yes	No
3. Blakeman et al. [21]	UK	NPT	Chronic kidney disease management (telephone support)	NICE guideline [151]	Yes	Process evaluation	Qualitative	Prospective	Yes	Yes	No
4. Blickem et al. [22]	UK	NPT	Self-management support for long-term conditions (telephone support)		Yes	Process evaluation	Qualitative	Prospective	Yes	Yes	No
5. Brooks et al. [23]	UK	NPT	Care planning (mental health)	Systematic review [152]	Yes	Intervention design	Qualitative	Prospective	Yes	Yes	Yes
6. Buckingham et al. [24]	UK	NPT	COPD management in primary care	Systematic review [153]	–	Feasibility study	Mixed	Prospective	Yes	Yes	N/A
7. Clarke et al. [25, 26]	UK	NPT	Stroke rehabilitation	Systematic review [154]	Yes	Process evaluation	Qualitative	Prospective	Yes	Yes	Yes
8. Coupe et al. [27]	UK	NPT	Collaborative care for depression		Yes	Process evaluation	Qualitative	Retrospective	Yes	Yes	Yes
9. Finch et al. [28]	UK	NPT	Cognitive behavioural therapy	Systematic review [156]	Yes	Feasibility study	Qualitative	Prospective	Yes	Yes	N/A
10. Furler et al. [29–31]	Australia	NPT	Diabetes management in primary care		Yes	Process evaluation	Qualitative	Prospective	Yes	Yes	No
11. Gabbay et al. [32]	UK	NPT	Debt counselling for depression in primary care	NICE guideline [178]	Yes	Process evaluation	Qualitative	Prospective	Yes	Yes	Yes
12. Gask et al. [33]	UK	NPM	Collaborative care for depression	Systematic review [155]	Yes	Process evaluation	Qualitative	Retrospective	Yes	Yes	No
13. Grant et al. [34, 35]	UK	NPT	Primary care prescribing	NICE guideline [157]	Yes	Process evaluation	Qualitative	Prospective	Yes	Yes	No
14. Godfrey et al. [36]	UK	NPT	Delirium prevention in hospital	Systematic review [158]	Yes	Process evaluation	Qualitative	Prospective	Yes	Yes	Yes
15. Hind et al. [37]	UK	NPT	Aquatic therapy for children with Duchenne muscular Dystrophy		Yes	Feasibility Study	Qualitative	Prospective	Yes	No	No
16. Hooker et al. [38–42]	Australia	NPT	Identifying women at risk of intimate partner violence		Yes	Process evaluation	Mixed	Prospective	Yes	Yes	Yes
17. Kennedy et al. [43–46]	UK	NPT	Social network support in long-term conditions	Systematic review [159]	Yes	Process evaluation	Qualitative	Prospective	Yes	Yes	Yes
18. Khowaja et al. [47]	India, Mozambique, Nigeria, Pakistan	NPT	Maternal health in low-income countries	WHO guideline [160]	–	Feasibility study	Mixed	Prospective	Yes	Yes	Yes
19. Leon et al. [48]	South Africa	NPM	Testing and counselling for HIV in South Africa	Systematic reviews [161–163]	–	Process evaluation	Qualitative	Retrospective	Yes	Yes	No
20. Mair et al. [49]	UK	NPM	Telemedicine for COPD	Systematic review [164]	–	Process evaluation	Qualitative	Retrospective	Yes	Yes	N/A
21. Ong et al. [50–52]	UK	NPT	Osteoarthritis guidelines in primary care	NICE guideline [165]	–	Process evaluation	Qualitative	Prospective	Yes	Yes	Yes
22. Ricketts [53]	UK	NPT	Chlamydia screening in primary care		–	Process evaluation	Qualitative	Retrospective	Yes	No	No
23. Speed et al. [54]	UK	NPM	Management of constipation in primary care		Yes	Process evaluation	Qualitative	Prospective	Yes	Yes	No
24. Sturgiss et al. [55]	Australia	NPT	Weight management programme in primary care		Yes	Feasibility study	Quantitative (survey)	Prospective	Yes	No	No
25. Thomas, L. et al. [56, 57]	UK	NPT	Stroke rehabilitation (incontinence)	RCP-ICSWP guideline [166]	Yes	Process evaluation	Qualitative	Prospective	Yes	Yes	Yes
26. Willis [58]	Australia	NPT	Community support for women with postnatal depression		Yes	Historical review of documents	Textual analysis	Retrospective	Yes	N/A	N/A

N/A not available

In Table 2, we show that NPT was employed in in 82 uncontrolled studies, and these generated 90/130 (69.2%) articles [59–148]. These included feasibility studies (*n* = 20) and process evaluations (*n* = 54), and seven were what we have called ‘field studies’ which focused on general conditions in which interventions might take place, rather than the progress of specific interventions. One study was an ethnography of a set of socio-technical practices [103]. Qualitative methods were used in 72 studies. Of the remainder, seven were mixed methods studies, two were surveys, and one was a prospective cohort study.

**Table 2:** Uncontrolled studies using NPT as their analytic framework

Study	Country of origin	Theory frame	Implementation problem	Evidence base cited to support intervention	Use of NPT specified in protocol	NPT study type	Data collected	Application of NPT to data	Factors leading to intervention success or failure	Differences between categories of participants	Differences between settings
27. Aarts et al. [59]	Netherlands	NPM	Infertility support (online)	Systematic review [167]	–	Process evaluation	Qualitative	Retrospective	Yes	Yes	N/A
28. Agbakoba et al. [60–62]	UK	NPT	Telecare/digital health in the community	Systematic review [168]	Yes	Process evaluation	Qualitative	Prospective	Yes	Yes	Yes
29. Alharbi et al. [63]	Sweden	NPT	Person-centred care		–	Process evaluation	Qualitative	Retrospective	Yes	Yes	N/A
30. Ahmed et al. [64]	UK	NPT	Screening questionnaire (genetic conditions in primary care)	Systematic review [169]	–	Feasibility study	Qualitative	Retrospective	Yes	No	No
31. Alverbratt et al. [65]	Sweden	NPT	Patient assessment tool in psychiatry		–	Process evaluation	Qualitative	Prospective	Yes	Yes	Yes
32. Ariens et al. [66]	Netherlands	NPT	Teledermatology		Yes	Process evaluation	Quantitative (survey using eHit Toolkit [226])	Prospective	Yes	No	No
33. Atkins et al. [67]	South Africa	NPM	Supporting treatment adherence in tuberculosis	Systematic review [170]	–	Process evaluation	Qualitative	Retrospective	Yes	Yes	No
34. Bamford et al. [68]	UK	NPT	Nutrition guidelines	FSA guideline [171]	Yes	Process evaluation	Qualitative	Prospective	Yes	Yes	No
35. Basu et al. [69]	UK	NPT	Improving motor outcome in infants after perinatal stroke			Feasibility study	Qualitative	Prospective	Yes	No	N/A
36. Bayliss et al. [70]	UK	NPT	Training for chronic fatigue management	NICE guideline [172]		Feasibility study	Qualitative	Prospective	Yes	Yes	No
37. Bee et al. [71]	UK	NPT	Cognitive behavioural therapy by phone	Systematic reviews [227, 228]		Feasibility study	Qualitative	Prospective	Yes	No	No
38. Bocum et al. [72]	Burkina Faso	NPM	Antenatal syphilis screening			Feasibility study	Qualitative	Retrospective	Yes	No	Yes
39. Bouamrane and Mair [73]	UK	NPT	Surgical assessment (online)	Systematic review [168]	Yes	Process evaluation	Qualitative	Prospective	Yes	No	N/A
40. Bouamrane and Mair [74]	UK	NPT	Electronic referrals (online)	Systematic review [168]	Yes	Process evaluation	Qualitative	Prospective	Yes	No	N/A
41. Bouamrane and Mair [75]	UK	NPT	Surgical assessment (online)	Systematic review [173]	Yes	Process evaluation	Qualitative	Prospective	Yes	Yes	N/A
42. Bridges et al. [76]	UK	NPT	Compassionate nursing care	Systematic reviews [76, 229]	Yes	Process evaluation	Qualitative	Prospective	Yes	No	Yes
43. Chiang et al. [77]	Australia	NPT	Risk assessment tools	Systematic review [174]	–	Feasibility study	Qualitative	Prospective	Yes	No	No
44. Conn et al. [78]	Canada	NPT	Improving recovery after colorectal surgery	Meta-analysis [175]	–	Process evaluation	Qualitative	Retrospective	Yes	Yes	No
45. Desveaux et al. [79]	Canada	NPT	Hospital accreditation	Systematic review [230]	–	Process evaluation	Qualitative	Retrospective	Yes	Yes	yes
46. Dickinson et al. [80]	UK	NPT	Cognitive stimulation for people with dementia			Process evaluation	Qualitative	Retrospective	Yes	Yes	Yes
47. Dikomiitis et al. [81]	UK	NPT	Decision support tool for cancer		–	Feasibility study	Qualitative	Prospective	Yes	No	No
48. Drew et al. [82]	UK	ENPT	Fracture prevention clinics	NICE guidelines [176, 177]	–	Process evaluation	Qualitative	Retrospective	Yes	Yes	No
49. Dugdale et al. [83]	UK	NPT	Substance misuse management (online)		–	Process evaluation	Qualitative	Prospective	Yes	Yes	No
50. Ehrlich [84]	Australia	NPT	Care coordination in long-term conditions		Yes	Field study	Qualitative	Prospective	N/A	N/A	N/A
51. Finch [85]	UK	NPM	Telecare/telemedicine		–	Field study	Qualitative	Prospective	Yes	No	No
52. Franx et al. [86]	Netherlands	NPT	Collaborative care for depression	NICE guideline [178]	Yes	Process evaluation	Qualitative	Retrospective	Yes	Yes	No
53. French et al. [87, 88]	UK	NPT	Stroke management using telecare	Systematic review [179]	Yes	Process evaluation	Qualitative	Prospective	Yes	Yes	No
54. Foss et al. [89]	Norway	NPT	Social network mapping for chronic disease management	Systematic review [231]	Yes	Process evaluation	Qualitative	Prospective	Yes	No	No
55. Foster et al. [90]	Australia	NPT	Diabetes management	Systematic review [180]	–	Feasibility study	Qualitative	Prospective	Yes	Yes	No
56. Gould et al. [91]	UK	NPT	Infection prevention and control		–	Process evaluation	Qualitative	Retrospective	Yes	Yes	No
57. Green et al. [147]	UK	NPT	Cancer risk assessment tool	NICE guideline [181]	–	Feasibility study	Qualitative	Retrospective	Yes	N/A	N/A
58. Gunn et al. [92]	Australia	NPT	Reorganisation of primary care mental health services	Systematic review [155]	–	Process evaluation	Qualitative	Retrospective	Yes	No	Yes
59. Hall et al. [93]	UK	NPT	Monitoring technologies in care homes for people with dementia	Systematic review [232]		Process evaluation	Qualitative	Retrospective	Yes	Yes	Yes
60. Hall et al. [94]	UK	NPT	Supporting staff working with people with autism		Yes	Process evaluation	Qualitative	Prospective	Yes	No	No
61. Hazell et al. [95]	UK	NPT	Guided self-help cognitive therapy	NICE guideline [233]	Yes	Process evaluation	Quantitative (survey)	Prospective	Yes	Yes	N/A
62. Henderson et al. [96]	UK	NPT	Diagnostic decision support in primary care	Systematic review [167, 182]	–	Process evaluation	Mixed	Prospective	Yes	No	N/A
63. Herbert et al. [97]	UK	NPT	Enhanced recovery after surgery			Process evaluation	Qualitative	Prospective	Yes	Yes	N/A
64. Hoberg et al. [98]	USA	NPM	Group therapy model	APA guideline [234]	–	Feasibility study	Qualitative	Prospective	Yes	No	No
65. Holtrop et al. [99]	USA	NPT (collective action constructs)	Care management for chronic disease in primary care		Yes	Process evaluation	Qualitative	Prospective	Yes	No	Yes
66. Kanagasundaram et al. [100]	UK	NPT	Diagnostic decision support (acute kidney injury)	NICE guideline [183]	–	Feasibility study	Mixed	Retrospective	Yes	Yes	N/A
67. Kulnik et al. [101]	UK	NPT	Inter-professional self-management support	Systematic review [184]	–	Process evaluation	Mixed	Prospective	Yes	Yes	Yes
68. Johnson et al. [102]	UK	NPT	Guideline implementation	Overview of systematic reviews [235]	Yes	Process evaluation	Quantitative (prospective cohort intervention)	Prospective	Yes	Yes	N/A
69. Jones, C. et al. [103]	UK	NPT	Diagnostic point of care testing		–	Ethnographic case study	Qualitative	Prospective	Yes	Yes	N/A
70. Jones, F. et al. [104]	UK	NPT	Self-care training programme for stroke practitioners		–	Process evaluation	Qualitative	Retrospective	Yes	No	No
71. Leggat et al. [105]	Australia	NPT	Quality improvement in hospitals	Systematic review [236]	No	Process evaluation	Qualitative	Retrospective	Yes	Yes	Yes
72. Lhussier et al. [106]	UK	NPT	Care planning in primary care		No	Field study	Qualitative	Retrospective	Yes	Yes	N/A
73. Ling et al. [107]	UK	NPT	Integrated care policy		–	Process evaluation	Qualitative	Retrospective	Yes	Yes	Yes
74. Lloyd et al. [108, 109]	UK	NPT	Shared decision-making tools	Systematic review [185]	Yes	Feasibility study	Qualitative	Retrospective	Yes	Yes	Yes
75. Lowrie et al. [110]	UK	NPT	Chronic heart failure management in the community	NICE guideline [186]	–	Feasibility study	Qualitative	Retrospective	Yes	Yes	N/A
76. Martindale et al. [111]	UK	NPT	Management of acute kidney injury in the community	NICE guideline [183]	–	Process evaluation	Qualitative	Prospective	Yes	Yes	Yes
77. May et al. [112]	UK	NPT	Telecare for chronic disease management in the community	Systematic review [164]	Yes	Process evaluation	Qualitative	Prospective	Yes	Yes	Yes
78. Morton and Wigley [113]	UK	NPT	Nursing assessment tool for maternal/child health in the community		Yes	Process evaluation	Qualitative	Prospective	Yes	No	N/A
79. Murray et al. [114]	UK	NPT	E-health systems	Systematic review [187]	Yes	Process evaluation	Qualitative	Prospective	Yes	Yes	Yes
80. Newton [115]	Australia	NPT	Caseload midwifery models	Systematic review [188]	Yes	Process evaluation	Mixed	Prospective	Yes	No	N/A
81. Nordmark et al. [116]	Norway	NPT	Discharge planning	Systematic review [189]	–	Feasibility study	Qualitative	Prospective	Yes	Yes	Yes
82. O’Connell and Kaner [117]	UK	NPT	Alcohol brief interventions in primary care		–	Field study	Qualitative	Retrospective	Yes	No	N/A
83. Owens and Charles [118]	UK	NPT	Text messaging in child and adolescent mental health services	Systematic review [190]	Yes	Feasibility study	Qualitative	Prospective	Yes	No	N/A
84. Polus et al. [119]	Australia	NPM	Chiropractic services for indigenous Australians		–	Feasibility study	Qualitative	Prospective	Yes	Yes	N/A
85. Pope et al. [120, 121]	UK	NPT	Decision support tools for emergency services		Yes	Process evaluation	Qualitative	Retrospective	Yes	Yes	Yes
86. Røsstad et al. [122]	Norway	NPT	Care pathways for older patients	Systematic review [191]	–	Process evaluation	Qualitative	Retrospective	Yes	Yes	No
87. Sanders et al. [123]	UK	NPT	Back pain management in primary care		Yes	Process evaluation	Qualitative	Retrospective	Yes	No	N/A
88. Scalia [124]	USA	NPT	Option Grid decision support tools	Systematic reviews [185, 237]	Yes	Field study	Qualitative	Prospective	Yes	No	Yes
89. Scantlebury [125]	UK	NPT	Maternity unit electronic health record	Systematic review [192]	Yes	Process evaluation	Qualitative	Prospective	Yes	Yes	N/A
90. Segrott et al. [126]	UK	ENPT	Adolescent substance misuse programmes	Systematic review [193]	Yes	Process evaluation	Mixed	Prospective	Yes	Yes	Yes
91. Shemeili [127]	Abu Dhabi	NPT	Medicines management in hospital care of older people		Yes	Process evaluation	Qualitative	Prospective	Yes	No	N/A
92. Shulver et al. [128]	Australia	NPT	Telecare for older people		Yes	Field study	Qualitative	Prospective	Yes	Yes	Yes
93. Spangaro et al. [129]	Australia	NPM	Screening for intimate partner violence	Systematic review [238]	–	Process evaluation	Qualitative	Retrospective	Yes	No	N/A
94. Stevenson [130]	UK	NPT	UK Clinical Practice Research datalink		Yes	Process evaluation	Qualitative	Prospective	Yes	No	No
95. Tarzia et al. [131]	Australia	NPT	Decision-making for older adults with dementia		–	Field study	Qualitative	Retrospective	Yes	Yes	N/A
96. Tazzyman et al. [148]	UK	NPT	Revalidation of medical practitioners		Yes	Process evaluation	Qualitative	Prospective (structured through the NoMAD Questionnaire)	Yes	Yes	N/A
97. Temple-Smith et al. [132]	Australia	NPT	Chlamydia testing in general practice		Yes	Process evaluation	Mixed	Prospective	Yes	No	No
98. Teunissen et al. [133–136]	Austria, England, Ireland, Greece, Netherlands	NPT	Migrant health		Yes	Process evaluation	Qualitative	Prospective	Yes	Yes	Yes
99. Thomas et al. [137]	Sweden	ENPT	Healthy lifestyle promotion in primary care		–	Process evaluation	Mixed	Retrospective	Yes	Yes	Yes
100. Tierney et al. [138]	Ireland	NPT	Interdisciplinary teams in primary care	Systematic review [194–196]	Yes	Process evaluation	Quantitative	Prospective	Yes	Yes	No
101. Toye et al. [139]	Canada	NPT	Assessment instrument for homecare		Yes	Feasibility study	Qualitative	Prospective	Yes	Yes	Yes
102. Trietsch et al. [140]	Netherlands	NPT	Quality improvement collaboratives	Systematic review [197]	–	Process evaluation	Qualitative	Retrospective	Yes	Yes	Yes
103. Vest et al. [141]	US	NPT	Clinical guideline implementation in chronic kidney disease	ACP guideline [198]	–	Process evaluation	Qualitative	Retrospective	Yes	N/A	N/A
104. Volker et al. [142]	Australia	NPT	Cardiovascular disease prevention		–	Process evaluation	Qualitative	Prospective	Yes	Yes	Yes
105. Webster et al. [143]	UK	NPT	Delivery of a psychosocial intervention for people with depression and long-term conditions		Yes	Process evaluation	Qualitative	Prospective	Yes	No	No
106. Walker et al. [144]	Australia	NPT	Colorectal cancer risk prediction	NICE guideline [199]	–	Feasibility study	Qualitative	Retrospective	Yes	No	No
107. Wilhelmsen et al. [145]	Norway	NPT	Web-based cognitive behavioural therapy	Systematic reviews [200, 201]	–	Feasibility study	Qualitative	Retrospective	Yes	No	No
108. Wilkes et al. [146]	UK	NPM	Open access infertility clinics		–	Feasibility study	Qualitative	Retrospective	Yes	Yes	No

N/A not available

### What was being implemented?

Studies included in this review fell into seven categories. The most numerous group of studies were those concerned with *service organisation and delivery* (*n* = 29, 26.9% [23, 27, 32–35, 43–46, 58, 76, 79, 82, 84, 86, 89, 91, 92, 99, 105–107, 110, 115, 116, 119, 122, 127, 133–136, 140, 146, 148]). For example, in the UK, Grant et al. [34, 35] evaluated a complex intervention aimed at reducing risk in prescribing in primary care. They used NPT in ‘identifying and describing the components and sub-components of the intervention’ to understand ‘the nuances associated with collective implementation’. The next most numerous group of studies focused on the implementation of *diagnostic and therapeutic interventions* (*n* = 28, 25.9% [19, 24–26, 28–31, 36, 37, 47, 48, 55–57, 67, 69, 78, 80, 90, 95, 97, 98, 103, 104, 111, 117, 123, 126, 137, 142, 143]). For example, in the USA, Hoberg et al. [98] examined the implementation of a new form of group therapy for people with mental health problems, while Leon et al. [48] showed how provider initiated testing and counselling for HIV was successfully normalised in a South African setting. Studies of implementation of *E-Health and telemedicine*—including telephone advice—were also numerous (*n* = 21, 19.4% [21, 22, 49, 59–62, 66, 71, 73–75, 83, 85, 87, 88, 93, 112, 114, 118, 125, 128, 130, 145]). Here, a Norwegian team led by Wilhelmsen et al. [145] showed how problems of participation and action—and especially the interactional workability—of a service providing internet-based cognitive behavioural therapy led to ambivalence on the part of general practitioners about its use, to low levels of follow-up and to doctors reverting to ‘standard treatment’ [145]. Less numerous (*n* = 11, 10.1%, were studies of the implementation of *screening and surveillance tools* [38–42, 53, 64, 65, 72, 77, 113, 129, 132, 139, 144]). In a feasibility study, Ahmed et al. [64] showed that integrating a family history questionnaire about common genetic diseases into the workflow of primary care was unlikely without significant changes to the pattern of GP-patient interactions, and these were unlikely to be supported by clinicians. Such professional factors also affected the outcome of studies of *decision support and shared decision­making* (*n* = 8, 7.4% [81, 96, 100, 108, 109, 120, 121, 124, 131, 147]). In this category, in the USA, Scalia et al. [124] compared the implementation and integration of decision support tools between two major healthcare systems. This study raised important questions about how the interactions between clinicians’ (micro-level) experiences of the workability of complex interventions and meso-level organisational processes through which reflexive monitoring mechanisms play out their effects. Some studies were also explicitly concerned with implementing *change in professional roles* (*n* = 7, 6.5% [20, 54, 63, 70, 94, 101, 138]). For example, Thomas et al. [56, 57] showed how changes in roles and workload interacted to promote the routine embedding of an intervention intended to manage incontinence in stroke patients. Finally, a small group of studies were concerned with *guideline implementation* (*n* = 4, 3.7% [50–52, 68, 102, 141]). Here, Vest et al. [141] described a study in the USA of the implementation of guidelines for the management of chronic kidney disease in primary care. They asserted that NPT could not only identify key barriers to practice but could also guide intervention choice.

### Was what was being implemented evidence-based?

Studies included in this review were mainly focused on reporting the implementation of complex healthcare interventions. Most of these studies had a translational component and made some claim about the evidence underpinning interventions. This evidence was heterogeneous and included qualitative studies [120, 121], implementation appraisals [133], meta-ethnographies [137] and previous trial results [38–41]. However, the most common appeal to an evidence base in studies included in this review was through references to systematic reviews and rigorously developed clinical guidelines. Across the studies included in the review, 64/108 (59.2%) were linked to such support by their authors [149–201]. As Tables 1 and 2 show, systematic reviews and rigorous guidelines were cited in support of 17/26 (65.4%) controlled studies and 47/82 (57.3%) uncontrolled studies.

### How did researchers justify the use of NPT?

As Tables 1 and 2 show, in 54/108 (50%) of the studies included in this review, the use of NPT appeared to have been planned in advance, and this was included in the study protocol. Amongst controlled studies, 19/26 (73%) of studies made explicit reference to including NPT in study protocols, while only 35/82 (42.7%) of uncontrolled studies did so. Not all papers offered a justification for using NPT. For the most part, authors characterised NPT as a conceptual framework that explains implementation processes and thus structures study design and data analysis. For example, Brooks et al. [23] justify it thus:

‘Normalisation Process Theory (NPT) has been used to consider complex interventions prior to the development of a randomised control trial to test their effectiveness (…). It has also been used in the context of mental health to explore the impact of new forms of collaborative care on the way in which professionals carry out their routines of work in primary care (…). The four constructs (coherence, cognitive participation, collective action and reflexive monitoring) permit a means of appraising factors that might ‘promote and inhibit the routine incorporation of complex interventions into everyday life’ (…). It focuses on the work that people need to do to ensure interventions become ‘normalised’. As a heuristic framework it can support the optimisation of a trial intervention at three points:

- supporting intervention design
- describing the context of a trial
- supporting the interpretation of a trial’s results’ [23].

Other papers reflected in more general terms on NPT’s empirical grounding (e.g. [28, 50, 52, 67, 73–75, 87, 114, 122]) and its usefulness in thinking about implementation design (e.g. [27, 33, 67, 106, 147, 202, 203]).

### Did NPT explain implementation outcomes?

In all but one study in the review [84], there was evidence that implementation outcomes could be explained by reference to the mechanisms specified by NPT. For example, Scalia et al. [124], state that their study

suggests that patient decision aids that are specifically designed for use in clinical encounters can be embedded in clinical settings, provided there is agreement about the *need* to use them, that the team members are willing to work together to make sure that such tools can be integrated in existing work patterns, and understood as making a positive overall contribution to the work that has to be performed. These considerations match the mechanisms of the NPT, which provides an explanatory framework for understanding the sustained use of these tools by the two systems examined. The motivation for the use of the Option Grid at Capital*Care* was their wish to achieve success in an external quality improvement initiative. At HealthPartners, implementation efforts were motivated by a ‘champion’ physician. The nursing staff also played a pivotal role by systematically identifying eligible patients and providing those patients with the relevant encounter tool. These organizations, in different ways and to different degrees, exhibited *coherence*, *collective action* and *cognitive participation* that supported the sustained use of the tools. The organizational appraisal, in other words, their *reflexive monitoring*, was positive overall, despite concerns about readability and time pressures.

(Part omitted)

Implementing patient decision aids into clinical settings is a difficult process (…) In the UK, an implementation program known as MAking Good Decisions In Collaboration (MAGIC) highlighted the need for an organizational *coherence*, i.e. a widely held and agreed understanding of SDM principles in order to facilitate the implementation of patient decision aids (…). Commitment at multiple organizational levels has been recognized as an important precondition for implementation (…). This lack of commitment was noticeable at the Capital*Care* sites that did not use patient encounter tools [124].

Differences between participant groups were characterised in 69/108 (64%) studies and between settings in 36/108 (33%) studies. For example, Clarke et al. [26] placed this in the wider context of levels of analysis.

‘This paper briefly considers implementation theories in respect of complex interventions and provides an overview of process evaluations to set the context for the study. We draw on Normalisation Process Theory (NPT) (…) as a conceptual lens through which to explore those features of the implementation process that were intended to secure practice change and to engage caregivers in the program. We also consider the interaction between influential macro and micro contextual factors that affected delivery by multi-disciplinary stroke unit staff and suggest that prior focus on generative mechanisms identified within NPT can be used to inform implementation processes within complex healthcare settings’ [26].

NPT thus characterises core elements of implementation processes and the factors that shape them, and using NPT enabled researchers to explain the ‘work’ that is involved in implementation. Implementation involves interactions between mechanisms and contexts that are highly complex and emergent. Dynamic elements of context can exercise powerful constraints on action. The sources of these constraints included system-level processes that structured behaviour (e.g. the role of fee for service payments in undermining the implementation of self-care programmes [43]) and micro-level conflicts within contexts (e.g. disagreements over participation and intervention legitimacy [20, 25]).

### How did researchers apply the theory’s constructs?

**Fig. 2:**
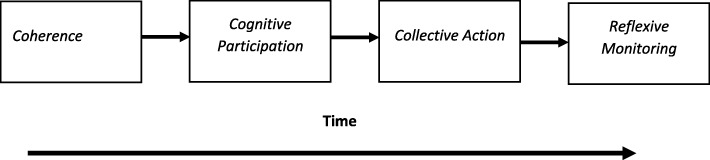
Interactions between NPT mechanisms: sequential operation over time (e.g. Alharbi et al. [63])

Implementation processes in NPT are explained by the operation of social mechanisms that motivate and shape collective action. Researchers using the theory employed its constructs in four distinctive ways, irrespective of the iteration of the theory that they used. We show examples of these diagrammatically below. First of all, some researchers clearly found it helpful to see the theory as describing a linear process in time [22, 63], in which the operation of mechanisms followed sequentially from each other (Fig. 2). In these studies, sense-making was seen as a necessary precursor to participation, and a degree of cognitive participation was required before collective action—in the form of an actual implementation process—could take place. Reflexive monitoring was seen as the final stage in the implementation process. However, research reported in this review often focused on feasibility studies or on the early stages of implementation life cycles in process evaluation. This skews their analyses towards the implementation phase of studies rather than their embedding and integration in everyday practice.

**Fig. 3:**
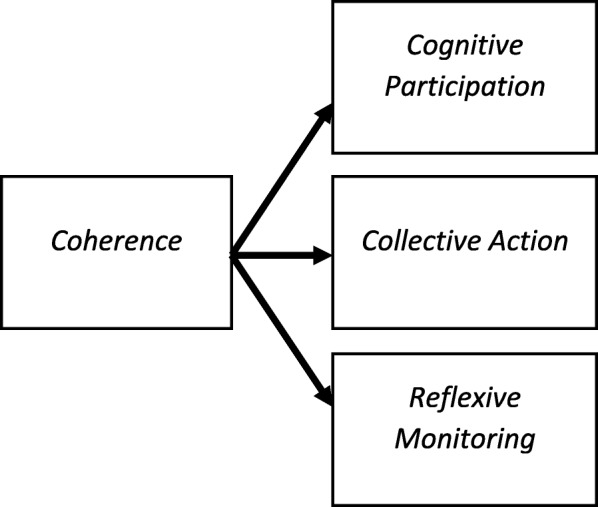
Interactions between NPT mechanisms: obligatory starting point (e.g. Finch [85])

**Fig. 4:**
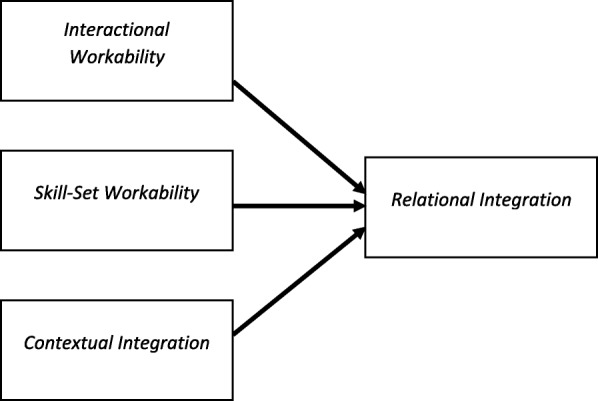
Interactions between NPM mechanisms: relational integration as an obligatory point of passage (Holtrop et al. [99])

As Fig. 3 shows, the focus on the ‘front end’ of studies leads to an emphasis on ‘coherence’ and sense-making work as an obligatory point of departure for implementation processes (e.g. [27, 31, 50, 52, 57, 74, 85, 108, 113, 123, 131, 203]), sometimes at the expense of other activities. Figure 4 suggests a novel analysis of the relationship between mechanisms. Holtrop et al. examined the operation of components of collective action in the restructuring of provider reimbursement. Their analysis emphasised the role of relational integration as a precondition for normalisation. In this context, the operation of one mechanism might be an obligatory point of passage for the others. Holtrop et al. [99] state that

‘We found that effective care management normalization required relationship development between practice providers and staff and the care manager. Since identification and referral of patients needing care management was key to care management happening at all, the practice personnel understanding and appreciating the care manager role through a relationship with the care manager was critical. This was captured well through the NPT collective action component of relational integration. We interpreted relational integration to be the professional relationship development that occurred when care manager, providers and practice staff work together and understand and appreciate each other’s roles and contribution to patient care. Although it is its own component in NPT, we found it to be more of an outcome that occurred when the other components worked well (contextual integration, skill set workability and interactional workability). (…) We found that when any of the other components were not in place, there was also a lack of development of trust around shared patient care. Since care management is a relationship rich endeavor, the lack of this relationship is a key factor in care management’s disuse’ [99].

**Fig. 5:**
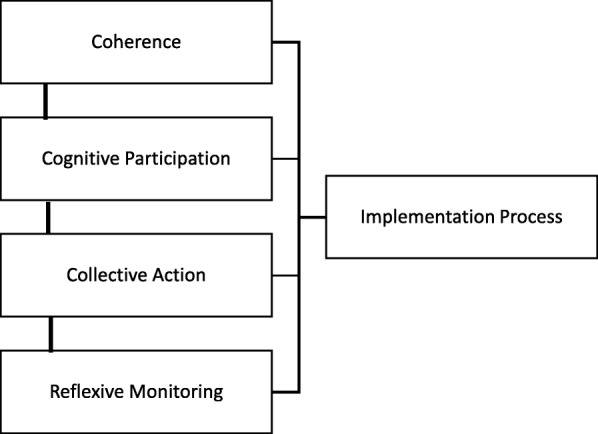
Interactions between NPT mechanisms: constant interaction between mechanisms (e.g. Hooker et al. [38–41])

Finally, as Fig. 5 shows, NPT assumes that its constituent mechanisms can operate simultaneously—but unevenly—rather than sequentially. Few studies in this review tracked the implementation of a complex intervention over its whole life. When they did, they tended to present summative rather than a formative accounts. An interesting example of a longitudinal study may be found in work by Tazzyman et al. [148] that depicted NPT in precisely these terms. They state that the mechanisms specified by NPT are

non-linear and interact dynamically to provide a comprehensive explanation of the implementation processes. NPT was designed to be applied flexibly, can be used at one or more points in a qualitative study, has been successfully used beyond its original field and provides a robust theoretical framework to understand the dynamics of implementation [148].

Tazzyman et al. [148] explored the processes that underpinned revalidation of medical practitioners in a qualitative study of senior decision-makers undertaken at three time points (2011, 2013 and 2015). They characterised respondents in their study in NPT terms as ‘sense-makers’ and then explored the process of implementing and embedding of revalidation as a broad policy initiative. They state that their contribution

has been to extend the use of NPT to explore the implementation of a broad and complex policy, with wide ranging implications for an entire profession, and the wider healthcare system. Much previous work using NPT in healthcare has addressed the implementation of micro level interventions. This expanded application of NPT has highlighted a number of factors which seem to have affected the implementation of revalidation. The four dimensions of the framework (see Table 3) had an intuitive relevance and provided a useful explanatory framework for understanding the implementation of revalidation. There is scope to apply NPT more widely to complex social interventions and policy initiatives at the organisational and system level in future [148].

More usually, longitudinal studies using NPT were process evaluations embedded in large complex intervention trials. As we have noted above and elsewhere [10], these permitted a more structured analysis of implementation processes and their motivating mechanisms over time [29–31, 38–41, 43–46, 50–52].

**Table 3:** Alverbratt et al. operationalise all constructs and subconstructs of NPT [65]. (Reproduced from the *Journal of Hospital Medicine* published under Creative Commons Attribution (CC-BY) licence)

Coherence ‘The significant qualities DLDA)	Cognitive participation ‘Enrolment and engagement of individuals and groups’	Collective action ‘Interaction with already existing practices’	Reflexive monitoring ‘How a practice is understood and assessed by actors implicated in it’
Differentiation. Understanding the difference between DLDA and ‘the old fashioned way’ of working in a psychiatric nursing context.	Initiation. The participants’ motivation in trying to incorporate the DLDA Tool.	Interactional workability. Operating DLDA.	Systematisation. The participants’ judgement of DLDA regarding usefulness and effectiveness.
Communal specification. The process through which users through teamwork share and create an understanding of this new practice.	Enrolment. The work participants do to organise themselves and their co-workers in the practice of DLDA.	Relational integration. Participants understandings of DLDA not only being aware of how and when to use DLDA, but also understanding the expressions of other staff members.	Communal appraisal. Communal appraisal regarding the outcomes and values of DLDA.
Individual specification. The process in which users create an understanding of the new practice.	Legitimation. The belief that DLDA is right for the context in terms of being a needed complement to existing tools and approaches.	Skill-set workability. Refers to how DLDA is conducted and distributed. This will influence how the work is defined and divided between participants.	Individual appraisal. Individual appraisal regarding the outcomes and value of DLDA.
Internalised meaning. The coherence of DLDA was based on the meaning users collectively invest in it.	Activation of DLDA. What the participants could do together to improve conditions for DLDA to be sustained and become part of daily practice.	Contextual integration. The incorporation of DLDA into a social context of the current wards.	Reconfiguration. Suggestions from participants that aim to modify and enhance the utility of the DLDA Tool.

### How did researchers integrate NPT into their research methods?

Researchers used two main strategies to translate the constructs of NPT into practically useful analytic tools. Some used deductive strategies that relied on framework or directed content [204, 205] analyses and in which interpretation of data was structured prospectively by the theory. These approaches often took the form of relating data to matrices of varying degrees of complexity. In Table 3, we show how Alverbratt et al. [65] have created a detailed matrix in which they reinterpret and operationalise all constructs and sub-constructs of NPT. This partly replicates the way that these were originally characterised in May and Finch’s account of NPT [7]. This approach defines and sets out all of the constituent elements of the work that drives implementation processes and permits data collection and coding using framework, or directed, content analysis [205]. The approach taken by Alverbratt et al. focuses on translating the content of the theory into practical research questions in a very precise way. Others focused on the main constructs of the theory prospectively, but within a more flexible framework. In Table 4, we show how Røsstad et al. [122] set out a matrix that links theory constructs to a description of data collected and in Table 5, we show how Nordmark et al. offer an even simpler data matrix, in which core constructs are linked to data collection opportunities [116]. Tazzyman et al. [148] used an analytic approach included both deductive and inductive elements.

A coding framework was developed using the four domains and sub-domains of NPT by using an adapted version of the NoMAD instrument (part omitted), which was developed to assess implementation processes (Normalization Measure Development is an instrument designed for assessing the implementation of complex interventions). The adapted NoMaD instrument was applied to the transcripts by coding evidence of the sub-domains in Dedoose [206]. Following coding, two members of the research team (AT and JF) analysed the data across the three interview stages, using the constant comparative method, in order to understand changes and continuities over time. The inductive method of constant comparison analysis involved searching within individual transcripts, making comparison between transcripts within the same cohort, and comparing transcripts from different cohorts for conceptual similarities and differences. This method was combined with the deductive approach of using the four domains on NPT as a framework for the analysis.

Tazzyman et al.’s hybrid approach enabled them to develop a theory-led analysis, without needing to force data into a rigid theoretical framework. However, many studies took a more straightforward inductive approach to data collection and analysis. When studies collected and analysed qualitative data inductively—in the *light of NPT*—rather than deductively using framework approaches, there was less pressure on them to interpret their qualitative data within an inflexible coding framework. For example, in Table 6, we show how Bamford et al. [20] described the ways that their inductively generated data categories mapped on to NPT constructs. This group of papers includes a group of highly illuminating studies across the life course of complex intervention trials. Bamford et al.’s [20] process evaluation of the CAREDEM trial, and Kennedy et al.’s [43–46] account of the WISE trial explain how structural factors militated against processes of cognitive participation. In their longitudinal accounts of the MOVE [38–41] and STEPPING-UP [29–31] Trials, Hooker et al. and Furler et al. show how mechanisms of coherence, cognitive participation and collective action interact to support the embedding of complex interventions in practice. Importantly, these studies also showed that the intervention remained in play once the trials themselves had concluded.

**Table 4:** Røsstad et al. link constructs to data and compare sites [122]. (Reproduced from *BMC Health Services Research*, published under a Creative Commons Attribution (CC-BY) licence)

	Municipalities					
	A	B	C	D	E	F
	PaTH in use in full scale^a^		Elements of PaTH in use^a^		PaTH not in use^a^	
Makes sense (coherence^b^)						
Expecting PaTH to be useful	Yes	Yes	Yes	Yes	Yes	Yes
Regular staff understood how to use PaTH	Mixed	Mixed	Mixed	Mixed	Mixed	Mixed
Commitment and engagement (cognitive participation^b^)						
Sustained leadership	Yes	Yes	No	No	No	No
Practice in using checklists	Intensive	Intensive	Minimal	Minimal	Minimal	Minimal
General attention to PaTH at workplace	Yes	Yes	No	Nurses only	No	No
Facilitating use of PaTH (collective action^b^)						
Extra personnel resources	Yes	Yes	No	Yes	No	No
Major competing priorities	No	No	No	No	Yes	Yes
Usability in electronic health record	Good	Fair	Poor	Poor	Poor	Poor
Working schedule facilitated for PaTH	Yes	Yes	No	No	No	No
Checklists incorporated in daily routines	Yes	Yes	No	No	No	No
Value of PaTH (reflexive monitoring^b^)						
Impact on collaboration with the hospital	Mixed	Mixed	No	No	No	No
Impact on collaboration with GPs	Yes	Yes	No	Yes	No	No
Impact on service quality	Yes	Yes	No	Yes	No	Yes
Value for individual nurse/nursing assistant	Yes	Yes	No	No	No	No
Valued as a management tool	Yes	Yes	No	Yes	No	No

^a^Assessed 24 months (B–F) and 32 months (A) after introduction of PaTH in the municipalities

^b^Core constructs of the Normalization Process Theory

**Table 5:** Nordmark et al. link NPT related questions to a data matrix [116]. (Reproduced from *BMC Medical Informatics and Decision-Making* under a Creative Commons Attribution (CC-BY) licence)

	Coherence	Cognitive participation	Collective action	Reflexive monitoring
	What is the process?	Who performs the process?	How does the process get performed?	How is the process understood?
	How RNs, DNs and HCOs perceived the DPP and whether they experienced the DPP as valuable to them and agreed about its usefulness and purpose	Whether RNs, DNs and HCOs saw the DPP as a legitimate part of their work and whether they supported it over time	How the DPP was provided within the existing context, how the embedding and integration work had proceeded due to knowledge and resources	How RNs, DNs and HCOs individually and collectively evaluated the DPP and its supportive tools
	Factors that promote or inhibit the routine embedding of DPP.	Factors that promote or inhibit participation in DPP	Factors that promote or inhibit enacting DPP	Factors that promote or inhibit appraisal of DPP
Data source	No. of text units			
Survey	0	1	12	0
Interview RNs	0	119	225	78
Interview DNs, HCOs	0	122	80	59
Adverse events/information system failures	0	3	2	0
Workshops	12	8	37	6

**Table 6:** Bamford et al. [20] retrospectively map inductively generated themes onto NPT constructs. (Reproduced from *BMC Health Services Research*, published under a Creative Commons Attribution (CC-BY) licence)

Mapping of overarching themes and subthemes to NPT framework		
NPT construct	Theme	Subthemes
Coherence	Making sense of the case manager intervention	Perceived value of the concept of case management. Clarity over the case manager role.
Cognitive participation	Investment in case management	Practice investment in case management. Investment by case managers. Fit of case management with existing skill sets.
Collective action	Implementing case management in practice	Time available for case management. Implementation in research vs clinical practice. Support and supervision of case managers.
Reflexive monitoring	Appraising and embedding of case management	Assessing the impacts of case management. The ‘right’ intervention but at the wrong time. Embedding case management in practice.

### How did users’ criticise NPT

Critique of NPT as a theory was rare amongst the papers included in this review. However, it was not absent. For example, Clarke et al. [26] criticised an over-emphasis on agency at the expense of implementation contexts in NPT.

‘While May *et al* (…) acknowledge that the NPT generative mechanisms are in dynamic interaction with local contexts and external drivers, the framework primarily addresses the mechanisms. Indeed, the theory tends to place undue emphasis on individual and collective agency without explicitly locating this within, and as shaped by, the organisational and relational context in which implementation occurs’ [26].

Segrott et al. [126] take this further. They point to what they perceive as a focus on the agency of those involved in implementation, as opposed to those who experience the effects of that agency.

‘ENPT places considerable emphasis on the notion of implementation as an expression of agency. However, the agents in question appear to be mainly conceptualised as professional practitioners (e.g. nurses), rather than the participants who receive interventions. There is scope to consider further how the key constructs of ENPT can be applied to understand how participant (and non-participant) agency may shape whether interventions become integrated and embedded within delivery systems’ [126]

Beyond this, Alharbi et al. [63] criticised NPT for presenting a normative model of implementation that paid insufficient attention to idealised temporal aspects of implementation, a point echoed by Alverbratt et al. [65]. Critique was more often about the interaction between theory and method. Some articles (9/108) observed that NPT constructs overlapped, that the technical vocabulary of the theory was difficult and that as a result coding qualitative data was difficult [39, 44, 48, 59, 64, 67, 82, 99, 207]. Problems of this nature seemed less evident when researchers used a more inductive approach to qualitative data analysis (e.g. [25, 26, 38–41]) than they did when authors employed a framework approach (e.g. [39, 99]).

## Discussion

### Key results of the review

In this review, we identified 108 discrete studies of complex healthcare interventions and related implementation processes. These studies were reported in 130 journal articles published after 2008. In papers included in this review, researchers collected and analysed their data in ways that effectively provided a basis (i) for intervention design and implementation planning and (ii) for understanding the dynamics of implementation, embedding and integration. Three key results of the review are as follows:

- NPT appears to accurately depict important elements of implementation processes, and the constructs of the theory can be applied in a stable and consistent way within and between studies.
- NPT has provided conceptual tools for a large body of feasibility studies and process evaluations of complex healthcare interventions. It has successfully explained the outcome of such intervention studies.
- NPT can be applied flexibly and can be understood and mobilised by researchers and practitioners with diverse professional backgrounds, working across a variety of healthcare settings.

The use of NPT has coalesced around two main types of study: feasibility studies and process evaluations. However, unlike McEvoy et al.’s [17] review of NPT studies, we found that authors were justifying their choice of theory, and NPT was more frequently embedded in study protocols and thus being operationalised prospectively. However, concerns raised by McEvoy et al. about the lack of prospective application do not just apply to NPT. For example, Kirk et al. [18] point to the problem of low levels of *prospective* use of the CFIR [208] and PARIHS [209] frameworks. They point to the additional problem of lack of integration of theory into implementation research. Against this background, our review suggests that—although some authors have experienced difficulty with NPT’s technical vocabulary—users of NPT appear to be able to operationalise its concepts in consistent, stable ways to inform their work, and we can see evidence of theoretical integration in four kinds of studies.

i. Studies constructed with NPT in mind that reflect its characterisation of implementation processes in both intervention and evaluation design (e.g. Furler et al. [29–31]).
ii. Studies that used NPT constructs as sensitising devices to form questions about implementation processes, and then related their conclusions back to the predictions of the theory (e.g. Grant et al. [34, 35]).
iii. Studies that collected and analysed data inductively in the light of NPT and then developed an analysis of the ways that different mechanisms work to motivate and shape implementation processes. The major papers by Clarke et al. [34, 35] and Hooker et al. [38–41] are important examples of such work. So too are Kennedy et al.’s accounts of the WISE trial [43–46].
iv. Studies that treated qualitative data deductively and used prescheduled coding matrices for framework or directed content analysis. Nordmark et al.’s work [116] offers an example of the way that this approach to theory driven analysis can be handled without ‘fitting’ or ‘shoehorning’ data in a rigid way (see MacFarlane and O’Reilly-de Brún [210] on techniques to manage this problem in qualitative research).

These different approaches to mobilising theory suggest that NPT’s users have developed flexible explanatory strategies, and we have pointed to some of these in Figs. 2, 3, 4 and 5. In earlier papers [5–9, 11, 12], we have argued that theories are conceptual toolkits that can be used flexibly to deal with practical problems. This means that there is no definitive ‘right way’ to employ NPT. It can be used on its own or in combination with other theories in ways that are locally defined to solve problems in intervention design and evaluation.

### Limitations of this review

This review contributes to the literature on the incorporation of theory in implementation research, the benefits of this incorporation and the problems that can arise as a result. There are, of course, limitations to the review. Searches were undertaken in two databases, so it is possible that some studies were missed. It is questionable whether this would have altered the main findings and conclusions. Because Google Scholar is a search engine, and not a database, results of searches using it were not stable. Searches on Google Scholar also identified multiple versions of the same reference (e.g. versions of the published paper on publisher’s websites, records on institutional repositories, versions on personal websites and on academic social media sites such as Academia.edu and ResearchGate.Com). Sorting these involved significant additional work. It did however identify about 20 papers that would not otherwise have been included in the review. One paper was brought to our attention before appearing in any databases; however, subsequent updated searches did identify this paper. Equally, we excluded studies published in languages other than English, although there is some evidence that this is unlikely to be a major limitation [211].

We deliberately followed a two-step approach to data analysis, first identifying and characterising the use of NPT in implementation research, and then exploring the contribution made by NPT to understanding the dynamics of the processes of implementation and integration, and the limitations of its use. The characterisation is likely to be replicable by another team, but it is possible that a different group of researchers, with different backgrounds and different prior experience of NPT, would reach different conclusions. We have maximised the robustness of our findings by following a transparent process for analysis, including NPT-naïve researchers in the team, and holding frequent discussions amongst the team during the analysis. Finally, we made a deliberate decision to focus solely on the health care literature, and in light of this decision, our findings only apply to research on implementation in health care.

### Next steps for NPT development and empirical research

Most papers in the review used the elaboration of NPT published by May et al. [7, 8] in 2009. More recent iterations of the theory have focused on (a) the important role that social structural and social cognitive features of context play in mobilisation for implementation [9] and (b) the ways in which implementation processes demand that their participants negotiate with other actors and elements in the context in which they are set [10]. In these papers, we have already gone some way to answering the critique of Clarke et al. [26] on the relationship between agency and context. The critique offered by Segrott et al. [126], however, focused on the experiences of different groups of actors in implementation processes. They saw NPT as primarily being about the agency of professionals, rather than the experiences of patients and other participants in implementation processes. NPT both can be, and is, applied to those groups. We have developed theory in this area to explore the relationship between the implementation of complex interventions and *burden of treatment* (e.g. [212–215]), and there is now a discrete body of primary research literature (e.g. [216–220]) and systematic reviews (e.g. [221–223]) that utilises these theoretical perspectives to understand patient and caregiver experience.

NPT has developed iteratively. Future work to develop it will explore variations in the ways that NPT mechanisms motivate and shape implementation processes across and between settings, and between micro, meso and macro levels of activity. This will engender a comprehensive ‘whole system’ approach to understanding implementation processes. Future empirical research will also help us to explore and test the hypothesis that collective action mechanisms operate cumulatively and that some mechanisms are more significant than others in determining implementation process outcomes. Rigorous quantitative research will assist in this, but until recently, there has been no robust instrument through which quantitative investigations of NPT mechanisms could be done. However, the NoMAD instrument is now available to perform this task [224]. This will make possible both large-scale and comparative quantitative and mixed methods studies that will provide important insights into the role of NPT mechanisms and the form and direction of implementation processes. This should lead to rigorous statistical models of NPT mechanisms at work and so to new insights about implementation processes. Finally, despite attempts to make NPT more user friendly through the development of explanatory toolkits, some users have difficulty with its technical vocabulary. NPT training packages are now coming on stream that will help to solve this problem [225].

## Conclusion

Normalization Process Theory appears to offer its users a coherent and stable set of explanations of implementation processes. It characterises the mechanisms that motivate and shape these processes and so can be used to aid intervention development and implementation planning as well as evaluating and understanding implementation processes themselves. In particular, NPT appears to have offered a valuable set of conceptual tools to understand the dynamics of implementation within clinical trials. In the future, it will be important to connect collective action much more closely to context in implementation studies. Equally, it will be important to develop longitudinal and genuinely mixed methods studies. These will help us understand not only the dynamics of implementation but also variations in implementation, embedding and long-term integration and sustainability over time and between settings.

## Additional file

Additional file 1: Appendix data extraction tool. (PDF 156 kb)

## Abbreviations

ACP: American College of Physicians
APA: American Psychiatric Association
CAREDEM: Collaborative cARE for people with DEMentia in primary care (trial acronym)
COM-B: Capability + Opportunity + Motivation → Behaviour Change Model
COPD: Chronic obstructive pulmonary disease
Dedoose: Proprietary qualitative analysis software
DLDA: Daily life dialogue assessment
DN: District nurse
DPP: Discharge planning process
ENPT: Extended Normalization Process Theory
FSA: Food standards agency (England and Wales)
HCO: Homecare organiser
ICSWP: Inter-collegiate stroke working party (UK)
MOVE: Improving maternal and child health nurse care for vulnerable mothers (trial acronym)
NICE: National Institute of Health and Care Excellence
NoMAD: Normalisation of complex interventions—measure development
NPM: Normalization Process Model
NPT: Normalization Process Theory
PARIHS: Promoting action on research implementation in health services
PaTH: Patient trajectory for Home-dwelling elders
RN: Registered nurse
STEPPING-UP: Theory based change in practice systems and roles of health professionals in the primary care diabetes team (trial acronym)
WISE: Whole system informing self-management engagement (trial acronym)
