# Supplementary material for: Using Normalization Process Theory in feasibility studies and process evaluations of complex healthcare interventions: a systematic review
Source: Implement Sci. 2018 Jun 7;13:80. doi: 10.1186/s13012-018-0758-1 (PMC5992634; doi:10.1186/s13012-018-0758-1)
Supplement: Supplementary file 1 — Appendix data extraction tool. (PDF 156 kb) [file 13012_2018_758_MOESM1_ESM.pdf]

# NPT systematic review

Data extraction instrument: The uses and limitations of theory in Implementation Research: a systematic review of Normalization Process Theory applied to complex healthcare processes and interventions

## 1. Reviewer

- ○ AC
- ○ TF
- ○ MG
- ○ FM
- ○ CM
- ○ EM
- ○ MM
- ○ TR

## 2. Full citation

## 3. What iteration of the theory is used?
## Tick all that apply

- ☐ NPM (May 2006)
- ☐ NPT (May & Finch 2009)
- ☐ eNPT (May 2013)
- ☐ Not clear

Selected constructs (please detail)

## 4. Country of origin

- ○ England
- ○ UK
- ○ Ireland
- ○ Netherlands
- ○ US
- ○ Canada
- ○ Australia
- ○ Germany
- ○ South Africa

Other (please specify)

## 5. Study setting

- ☐ Community / Population
- ☐ Nursing Home / Residential Care
- ☐ Primary Care / General Practice
- ☐ Hospital / Secondary Care

Other (please specify)

## 6. Study type

- ☐ Development of an intervention
- ☐ Optimisation of an intervention, including formative evaluation of e.g. acceptability and feasibility
- ☐ Evaluation of effectiveness, through e.g. RCT or other method (to be described)
- ☐ Implementation of an intervention, to find out what the impact of the intervention is in routine use
- ☐ Implementation of an intervention, to find out how to get the intervention into routine use

Other (please specify)

## 7. Intervention type

- ◯ Diagnostics/Therapeutics
- ◯ Decision support/shared decision-making tool
- ◯ Screening/Surveillance
- ◯ Guideline/Evidence implementation
- ◯ New professional roles
- ◯ New patient/service user roles
- ◯ Informatics/Telemedicine/EHRs
- ◯ Organization/Delivery of care/pathway
- ◯ Policy change

Other (please specify)

## 8. Which is the PRIMARY intervention (if more than one)?

## 9. Sample selection

- ☐ Structured
- ☐ Purposive
- ☐ Convenience
- ☐ Not described
- ☐ Not clear

Other (details)

## 10. Sample size

- ☐ Unknown

N =

## 11. Study duration

- ◯ Not described

Described n= months

## 12. Sample composition

- [ ] Policy-makers
- [ ] Managers
- [ ] Professionals
- [ ] Patients/service users
- [ ] Not described

Other (please specify)

## 13. Method of data collection

- [ ] Qualitative interviews
- [ ] Qualitative observation
- [ ] Qualitative interviews + observation
- [ ] Documentary analysis
- [ ] Survey interviews
- [ ] Online or postal survey
- [ ] Mixed methods
- [ ] Other quantitative method
- [ ] Audit of routinely collected clinical data

Other (please specify)

## 14. If MIXED METHODS, please specify

## 15. How was NPT used?

- [ ] Prospectively, to inform research questions and study design
- [ ] Retrospectively, to analyse data already collected (framework or similar analysis)
- [ ] Retrospectively, to interpret data analysed by other means (e.g emergent or ‘thematic’ coding)

Other (please specify)

**16. Was the NPT Toolkit used to provide a framework for data collection and analysis?**

- [ ] Yes
- [ ] No

## 17. Do the authors provide a rationale for using NPT?

- [ ] yes
- [ ] No

Details

## 18. Do the authors discuss other NPT studies?

- [ ] Yes
- [ ] No

Details

## 19. Do the authors compare NPT with other theories?

- [ ] Yes
- [ ] No

Details

## 20. Do the authors identify limits or problems associated with NPT?

- [ ] Yes
- [ ] No

Details

## 21. Does the paper explicitly discuss NPM/NPT/eNPT constructs?

- [ ] Yes
- [ ] No

List constructs here:

**22. What is NPT used to explain?**

**23. What explanations are offered?**

**24. What are the overall conclusions of the paper?**
